# Users prefer generative AI to reflect real over idealized gender proportions

**DOI:** 10.1038/s41598-026-66305-7

**Published:** 2026-08-11

**Authors:** Florian Richter, Sebastian Krügel, Matthias Uhl

**Affiliations:** 1 Catholic University of Eichstätt-Ingolstadt, Ostenstr. 26, 85072 Eichstätt, Germany; 2 University of Hohenheim, Schloss Hohenheim 1, 70599 Stuttgart, Germany

**Keywords:** Fairness, Gender representation, Image generation, Generative artificial intelligence, AI ethics, Philosophy, Psychology, Psychology, Science, technology and society

## Abstract

Generative artificial intelligence (GAI) systems must make decisions about how to represent gender in images of social categories. Different standards exist for evaluating whether such representations are appropriate: systems can reflect current statistical distributions (proportional equality), depict equal proportions (uniform equality), or actively promote diversity (substantive equality). While different authors apply different standards, what constitutes an appropriate representation from users’ perspective is rarely investigated systematically. Understanding user expectations is crucial. Our study therefore investigates users’ evaluations of gender representation in various social categories. In an experiment with 719 US participants, we find that uniform equality and substantive equality play little role in user evaluations. Users prefer a representation that reflects their perception of current reality. Our results show that users prefer statistical alignment.

**Supplementary Information:**

The online version contains supplementary material available at 10.1038/s41598-026-66305-7.

## Introduction

Generative artificial intelligence (GAI) systems are increasingly used to create visual representations of people in various social contexts. When prompted to generate images of CEOs, firefighters, nurses, or other social categories, these systems must decide about how to represent demographic characteristics such as gender. These representational choices raise important questions about fairness and appropriate design: Should AI-generated images reflect current statistical realities? Should they depict equal proportions of men and women? Or should they actively promote diversity by over-representing underrepresented groups?

These questions have gained public attention through high-profile cases. Google’s Gemini system, for instance, generated images of indigenous people in German World War Two uniforms, exposing “the tensions inherent in attempting to balance inclusivity with historical accuracy”^1^. For historically specific contexts, there are verifiable facts that make certain representations accurate or inaccurate. However, for generic prompts like “generate a picture of a CEO,” the relationship between accuracy and fairness becomes more complex. While statistical ground truth exists (e.g., approximately 10% of Fortune 500 CEOs are women), it is unclear whether reproducing these statistics in AI-generated images is desirable or whether alternative distributions would be more appropriate.

Evans et al.^2^ distinguish between truthfulness, honesty, and fairness in AI systems. A system is truthful when its output corresponds to external facts about the world, while it is honest when its output faithfully reflects its internal representations based on training data. Fairness, however, introduces normative considerations that may diverge from both. A distribution reflecting current statistical realities would be truthful to the world as it is, but this might conflict with normative ideals of fair representation. The central question is which distribution should be implemented, and according to which standard this should be determined.

As we review in Sect. 2, the literature on fairness in image generation discusses various approaches. Some researchers use demographic or labor statistics as benchmarks, treating deviations from current realities as biases^3,4^. Others adopt uniform equality as a standard, evaluating systems based on how closely they approach gender parity^5,6^. Still others discuss substantive equality approaches that would temporarily over-represent certain groups as a form of affirmative action^6,7^. While these different standards are acknowledged in the literature, they are rarely justified explicitly, and, crucially, users’ own conceptions of appropriate representation are seldom investigated systematically.

Understanding user expectations, however, is not merely a matter of customer satisfaction. Research shows that AI systems can influence users in the moral domain, both consciously and subconsciously^8^. Moreover, users perceive moral advice from large language models as more trustworthy than advice from human ethics experts^9^. If AI systems systematically represent social categories in ways that diverge from what users consider appropriate, these systems may inadvertently nudge users away from their own normative standards. If we want to keep humans genuinely in the loop of decision-making, rather than merely nominally, we need to understand what users themselves consider fair and appropriate upon reflection.

Our study, therefore, investigates which conception of fairness most closely reflects what users consider appropriate for the representation of gender in AI-generated images across various social categories. We conducted an experiment with 719 participants from the United States, representative regarding age, sex, and ethnicity. Participants expressed both their empirical beliefs about actual gender distributions in six social categories (CEO, firefighter, nurse, kindergartner, thief, and terrorist) and their normative beliefs about appropriate distributions in AI-generated images of these categories.

Our findings show that users’ normative beliefs align closely with their empirical beliefs about reality. Participants prefer AI-generated images to reflect their perception of current statistical distributions rather than to depict gender parity or to over-represent underrepresented groups. This pattern holds across different types of social categories, whether male- or female-dominated, whether positively or negatively connoted, and across different demographic groups, including both men and women and participants with varying levels of sexist attitudes. The much-discussed standards of uniform equality and substantive equality play little role in users’ evaluations of appropriate representation in our study.

These results have important implications for the design of generative AI systems. They suggest that user-centric approaches would need to consider empirical data on social realities when representing gender in social categories, rather than relying on alternative distributions based on idealized notions of equality. At the same time, our findings raise normative questions that extend beyond user preferences: While understanding what users expect is crucial for informed user-centric system design, it does not automatically determine whether this is ethically appropriate.

The paper proceeds as follows. Section  2 reviews the literature on fairness in image generation, identifying three main approaches: proportional equality (based on demographic statistics), uniform equality (based on gender parity), and substantive equality (based on affirmative action). Section  3 describes our research design and methodology. Section  4 presents our results. Section  5 discusses the theoretical and practical implications of our findings and acknowledges important limitations.

## Related work

As outlined in the introduction, the question of how gender should be represented in AI-generated images of social categories can be approached from different normative perspectives. The existing literature on fairness in image generation reflects this plurality, employing various standards to evaluate whether representations are appropriate. For instance, a prompt like “Generate ten pictures of CEOs” could be modeled according to (1) the proportional distribution according to labor statistics (one of ten depicted persons is a woman), (2) according to gender equality or parity (five women are depicted), or (3) in accordance with an active affirmative action policy (ten women are depicted). Randomization has been discussed as well, e.g., for distributing organs^10^ or places at universities^11^. For image generation, it has been discussed by Hacker et al.,^1^. Nevertheless, they believe that it is not viable since it might be too complex, as the case of Googleʼs Gemini model showed by “over-diversifying historical representation”^1^.

Our focus here is on how these different conceptions of fairness have been operationalized in the literature on image generation. The following studies evaluate sensitive attributes by using clear benchmarks like demographic data or equal distribution (50/50) to check for biases in AI-based image generation automatically. Such formalized fairness definitions are typically not employed in user studies where users are mostly asked about what bias types they see in AI-generated outputs without presupposing any formalization of what they “would like” to see in these outputs. Thus, we excluded such studies from our review. In the following, we consider proportional equality, uniform equality, and substantive equality.

### Proportional equality: demographic statistics as ground truth

One strand of research postulates that demographic or labor statistics serve as the ground truth, and in these studies, generated images are compared to this benchmark. Any deviation from such statistics is considered unfair or biased. Mostly, gender is investigated across occupations. For instance, some took demographic data from the U.S. Bureau of Labor Statistics (BLS) and compared the generated images against this ground truth^3,4^. Naik and Nushi present for gender “an analysis of the representation of different occupations by DALLE-v2, relative to a baseline of labor statistics (i.e., the difference between BLS representation and model representation)”^12^. In another study, such data were used “to benchmark the prevalence of existing gender disparity by occupation”^13^. Generated images were also compared to demographic data, which was “collected from the American Society of Anesthesiologists (ASA) and the European Society of Anesthesiology and Intensive Care (ESAIC)”^14^. Others compared the generated individual and group images with Australian data, particularly by creating images of Australian undergraduate medical students^15^. Similarly, they undertook a study of generated individual and group images of pharmacists and compared them against Australian data^16.^ In both cases, the “[g]enerated images included a disproportionately high proportion of white male medical students, which is not representative of the diversity of medical students in Australia,”^15^, or “white men as pharmacists, which is not representative of the diversity of pharmacists in Australia today”^16^.

### Uniform equality: equal distribution (50/50), parity as ground truth

Another strand of research uses a uniform distribution (50/50) of gender as a fairness metric. These are typically evaluations or benchmarks that assess contexts beyond the category of occupations and are therefore based on uniform equality, as there is no fundamental standard that can be used for comparison, as is the case in labor market statistics. For example, spatial composition or facial expressions are examined. Zhang et al., for instance, “cluster the embeddings of questions and answers to create equally sized gender-based subsets”^17^. To detect social biases in generated pictures, Cho et al., “compute the overall bias distribution” by using “mean absolute deviation (MAD) that measures the distance between detected gender/skin tone category/attribute distributions and unbiased uniform distribution” (“0.5 for gender; 0.1 for skin tone”)^5^. Ghosh and Caliskan have observed that neutral prompts (like “person”) produce more images of men across countries, i.e., not an equally uniform distribution between genders^18^. It seems that they also presuppose an equal distribution (50/50) as their ground truth. Similarly, others have used 0.5 as “the measure of gender bias”^19^, or have “assumed statistical parity to be fair, i.e., that an equal proportion of female- and male-appearing images is desired”^6^.

### Substantive equality: quota or affirmative action

It has also been discussed that “[t]emporarily over-representing a certain attribute, e.g., in advertisements,” could “be desired as it can promote awareness and transparency for bias and discrimination concerning this attribute”^6^. Nevertheless, such idealized affirmative action policies are not yet present in research since they are taken more as part of political^6^ or societal measurements^7^ to overcome inequality. For instance, “substantive algorithmic fairness” is introduced as a political measurement by eliminating “social conditions of domination and oppression” through a structural and relational approach^7^. Such an approach is not directly engaging with algorithms, but rather with social and political structures to overcome inequalities. Radovanovic et al., have proposed “a post-processing technique, called fair additive weighting”^20^ to do a kind of algorithmic affirmative action for automated decision-making. For image generation, Google used an affirmative action policy to over-represent women or minorities. However, it had a critical outcome since it diversified images in a historically inaccurate way^21^.

Wachter et al., write that the term “substantive equality should not be confused with a requirement for positive action (affirmative action), for example a requirement to hire people because of their gender”^22^. They use substantive equality to yield a conditional demographic parity, which is a notion of algorithmic fairness to identify, for instance, adequate candidates from underprivileged groups^22^. However, it seems that some of the authors encourage in a later paper affirmative action policies: Hacker et al., mentioned that there are “subtler biases from inadequate representation of protected groups,” which include “genAI output that, while not discriminatory in a single instance, has discriminatory effects over time. For example, a genAI system may predominantly display white men when asked for examples of people in important jobs”^1^. Although they take it as an open question which fairness metrics should be used, they write that “we can, and should, ask for more diversity than what current models often offer”^1^ (Sandra Wachter, one of the authors of the paper, also points in an interview to this problematic issue of “gender bias in AI, including bias in image generators and text prediction, where AI is more likely to assume a male gender for professions like doctors, and a female gender for professions like nurses”^23^.) This might imply some kind of affirmative action policy to compensate for past and present exclusion from workplaces (Judith Jarvis Thompson) or to contribute to the common good “without doing violence to justice” (Thomas Nagel)^24^; as two pleas for affirmative action justify such a policy^24^.

So, different fairness standards regarding generative AI are discussed in the literature. As some approaches suggest, the final decision on which one should be used is more of a political and societal question. However, participatory research to systematically investigate the normative expectations of users has, to our knowledge, not been conducted.

## Research question and design

### Research question

We investigate which normative conception of fairness reflects most closely what participants consider appropriate for image generation by AI, particularly for the representation of gender in certain social categories. The dependent variable is therefore participants’ belief about the proportion that they consider to be the appropriate representation of women (or men) in AI-generated images in a given social category (i.e., their normative belief in that category). To understand their notion of an appropriate representation, we crucially also elicited their empirical belief about the actual proportion of women (or men) in that category. To avoid participants anchoring their normative beliefs in their empirical beliefs, we distracted them with a multitude of questions on epistemologically irrelevant empirical beliefs.

### Research design and procedure

Participants were recruited via Prolific. The sample was from the USA and was representative regarding age, sex, and ethnicity. We pre-registered the study on aspredicted.org (https://aspredicted.org/srpc-3jtp.pdf). Ethical approval for the study was obtained from the German Association for Experimental Economic Research e.V. Furthermore, all participants provided informed consent before participating in the study. The research was conducted in accordance with the Declaration of Helsinki. The manuscript does not include any information or images that could lead to the identification of a study participant. The images for the study were AI-generated.

We asked the participants for their empirical beliefs about the proportions of certain groups in the USA in several contexts, including the variables of interest (i.e., the distribution of gender in different social categories). In total, the participants were asked to answer 15 such statistical questions. Six of these questions were about participants’ empirical beliefs of the actual proportion of women (or men) in different social categories (such as “How many out of 10 firefighters are men?”). The social categories that we used in our study were CEO, firefighter, nurse, kindergartner, thief, and terrorist. The nine other statistical questions were not relevant to our research question and were added to mask the intention of the study (for instance, “How many out of 10 people suffer from depression?”).

In addition to their empirical beliefs in 15 different contexts, we elicited participants’ normative beliefs in one of the six social categories in a second stage of the experiment. All in all, we employed six experimental conditions, one for each social category. We had 719 participants who were randomly assigned to one experimental condition with equal probability (i.e., roughly 120 participants per social category). To elicit normative beliefs, we told the participants in each condition that they would see images generated by artificial intelligence and asked them to evaluate which representation of women (or men) they found appropriate. In total, the participants were shown 11 images, each depicting 10 persons in one specific social category. Each of the 11 images contained a different gender distribution, ranging from 0 to 10 women (or men). To control for potential order effects, participants were randomly assigned to one of two presentation orders. Half of the participants saw the images in an order where the proportion of women increased from 0 to 10, and the other half saw them in a decreasing order from 10 to 0.

We chose the social categories for our experiment to represent a variety of characteristics that we considered important. Four social categories were male-dominated (i.e., CEO, firefighter, thief, and terrorist) and two were female-dominated (i.e., nurse and kindergartner). Both of the female-dominated categories were positively connoted. To control for the specific connotation of a social category, two of the male-dominated categories were positively connoted as well (i.e., CEO and firefighter), while the other two categories were negatively connoted (i.e., thief and terrorist). This variation in moral connotation was a deliberate design choice, allowing us to investigate whether the moral evaluation of a category influences participants’ preferences for gender representation. To confirm our intuition regarding the specific connotation of a certain social category, we asked the participants after they saw the 11 images to evaluate the respective social category morally on a scale from 0 (“morally very negative”) to 6 (“morally very positive”). Importantly, this moral evaluation was collected on a separate screen after participants had already indicated their normative beliefs about gender representation, ensuring that their normative beliefs were not influenced by their moral evaluation of the social category itself. If the average moral evaluation in a social category was smaller than 3, the respective category was considered negatively connoted; otherwise, it was considered positively connoted.

At the end of the experiment, we additionally collected some demographic variables of the participants (e.g., their gender) as well as their attitude toward sexism based on the Modern Sexism Scale developed in^25^.

## Results

Table 1 shows the average moral evaluation and the average estimated proportion of men in each social category. As expected, two of the six categories were evaluated as clearly negative in moral terms (i.e., thief and terrorist). Three categories were evaluated as clearly positive (i.e., firefighter, nurse, and kindergartner) and one as neutral or slightly positive (i.e., CEO). The proportion of men in each of the social categories estimated by the participants also corresponded to the expected empirical gender distribution. Four of the six social categories are male-dominated according to the participants (i.e., CEO, firefighter, thief, and terrorist), and two categories are female-dominated (nurse and kindergartner). Thus, the intended experimental manipulation was successful in that we aimed to represent two positively evaluated, male-dominated categories (i.e., CEO and firefighter), two negatively evaluated, male-dominated categories (i.e., thief and terrorist), and two positively evaluated, female-dominated categories (i.e., nurse and kindergartner).

**Table 1:** Manipulation check.

	Observations	Moral evaluation	Estimated proportion of men
CEO	120	3.25 (1.56)	0.80 (0.13)
Firefighter	120	5.58 (0.71)	0.82 (0.12)
Nurse	119	5.38 (0.82)	0.24 (0.13)
Kindergartner	120	5.47 (0.89)	0.21 (0.14)
Terrorist	119	0.29 (0.81)	0.85 (0.17)
Thief	121	0.59 (1.01)	0.73 (0.16)

*Notes*: The table shows the number of observations, the average moral evaluation, and the average estimated proportion of men in each of the six social categories. Numbers in parentheses are standard deviations. The moral evaluation of a social category was rated on a scale from 0 (“morally very negative”) to 6 (“morally very positive”).

**Fig. 1:**
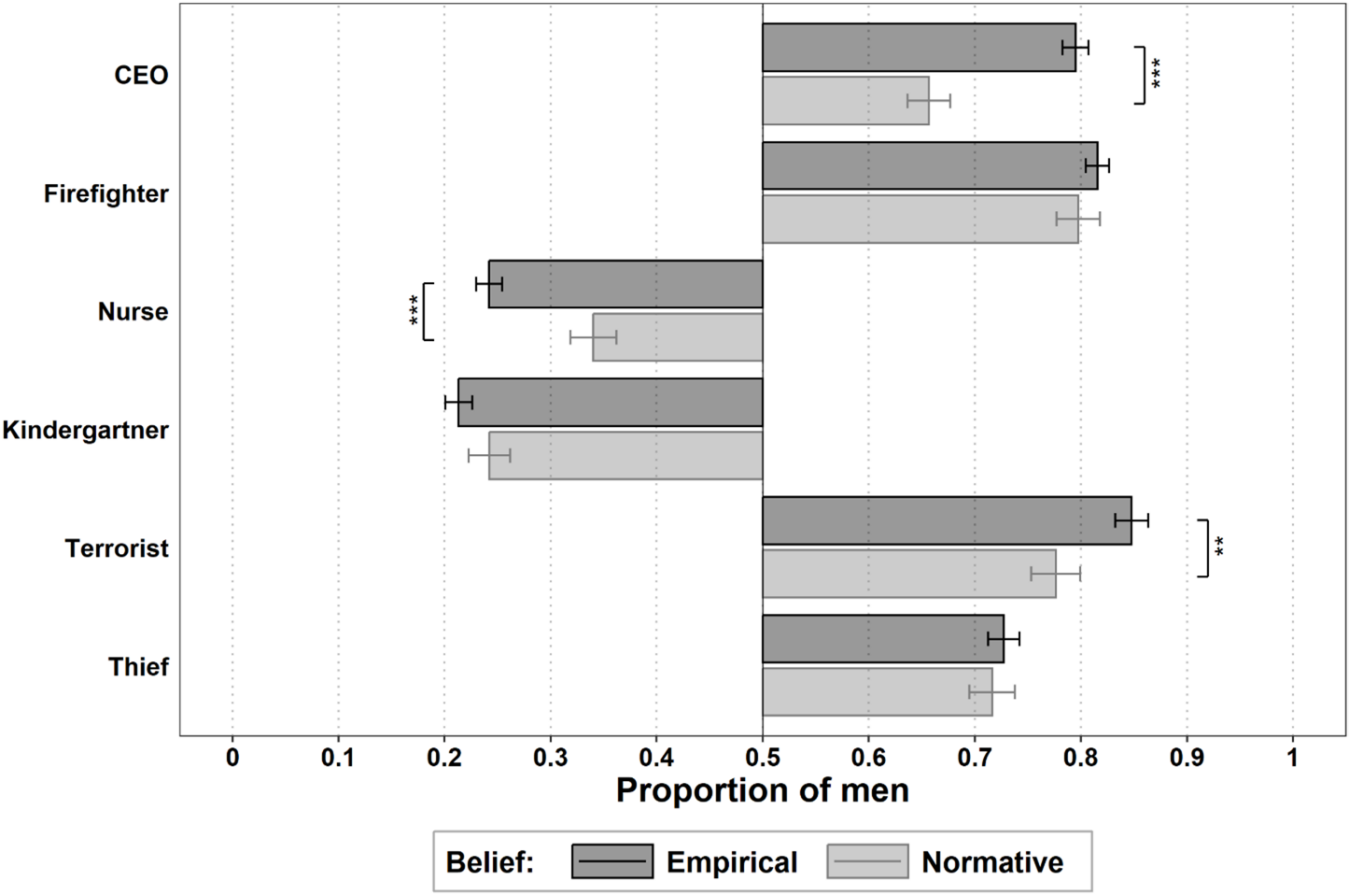
Participants’ empirical and normative beliefs about gender composition in AI-generated images in six social categories. *Notes*: The figure depicts means and standard errors of participants’ beliefs about actual (i.e., empirical) and appropriate (i.e., normative) proportions of men in six social categories. All significant differences between empirical and normative beliefs are reported in the figure. ^**^p < 0.01, ^***^p < 0.001.

Figure 1 compares participants’ average estimated proportion (i.e., their empirical belief) and average appropriate proportion (i.e., their normative belief) of men in each social category. Participants’ average normative belief deviates significantly from a gender-balanced distribution in each category (*|t| > 7.31*, *p* < 0.001 in each social category). This is evidence against a conception of fairness among participants that is based on uniform equality in AI-generated images. Since participants’ average normative beliefs always deviate from an equal gender distribution in the direction of the average empirical belief, regardless of whether the social category is male- or female-dominated, a substantive equality conception of fairness as a benchmark for AI-generated images is also not representative of participants’ attitudes.

Participants’ normative beliefs in all categories are closely aligned with their empirical beliefs, which suggests that proportional equality is the most likely fairness conception among our participants for AI-generated images. In three categories, participants’ normative and empirical beliefs are not even significantly different (*|t| < 1.54*, *p* > 0.12 in each of the three categories firefighter, kindergartner, and thief). In the other three categories, the participants’ normative beliefs deviate significantly from their empirical beliefs in the direction of equal gender distribution (*|t| > 2.83*, *p* < 0.01 in each of the three categories CEO, nurse, and terrorist). However, normative beliefs are again strongly influenced by empirical beliefs. Moreover, from the participants’ perspective, it does not seem to matter much whether the social category is male- or female-dominated or whether it has positive or negative connotations. None of these characteristics of social categories seem to influence normative beliefs systematically.

**Fig. 2:**
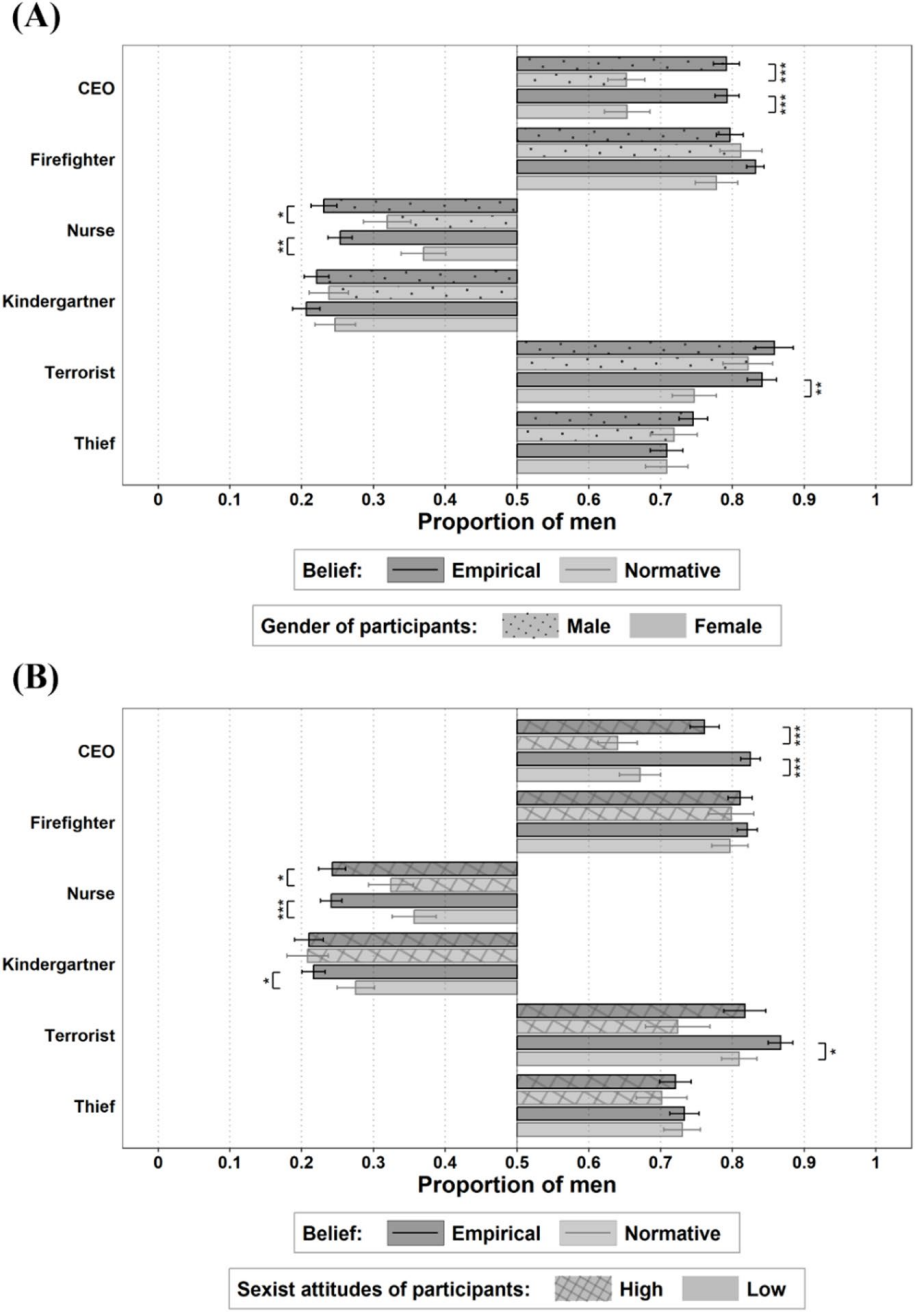
Participants’ empirical and normative beliefs about gender composition in AI-generated images in six social categories, separated by gender and sexist attitudes. *Notes*: The figure depicts means and standard errors of participants’ beliefs about actual (i.e., empirical) and appropriate (i.e., normative) proportions of men in six social categories. (**A**) shows beliefs separated by participants’ gender, (**B**) shows beliefs separated by participants’ sexist attitudes based on a median split. All significant differences between empirical and normative beliefs are reported in the figure. ^*^ *p* < 0.05, ^**^ *p* < 0.01, ^***^ *p* < 0.001.

Figure 2 shows that the patterns of results are very similar between men and women and between participants with low and high levels of sexist attitudes. Not only among men, but also among women, the average normative beliefs deviate significantly from a gender-balanced distribution in the direction of average empirical beliefs (see Fig. 2A), regardless of whether the social category is male- or female-dominated *(|t| > 5.49*, *p* < 0.001 for men and *|t| > 4.19*, *p* < 0.001 for women in each social category). The normative beliefs of neither men nor women, therefore, correspond to a conception of fairness based on either uniform or substantive equality. Normative beliefs are closely aligned with empirical beliefs in all social categories. This applies to both genders, even when a certain gender is factually underrepresented in a social category. These results suggest that proportional equality is participants’ predominant conception of fairness in AI-based image generation, irrespective of their gender. The greatest deviations between empirical and normative beliefs for both genders are in the categories of CEO and nurse (men: *0.14* and *0.09*, women: *0.14* and *0.12*, respectively). The results are similar when participants are divided into two groups based on a median split according to their sexist attitudes, with one group exhibiting low levels and the other high levels of sexist attitudes (see Fig. 2B). (The mean values of the sexism scores in both groups of participants, low vs. high levels of sexist attitudes, differ substantially: 16.2 vs. 33.4 on a scale between 8 and 56, with high values indicating high levels of sexist attitudes, t > 36.54, *p* < 0.001.) Once again, all average normative beliefs deviate significantly from a gender-balanced distribution (*|t| > 4.70*, *p* < 0.001 for low and *|t| > 5.12*, *p* < 0.001 for high levels of sexist attitudes in each social category). Even participants with low levels of sexist attitudes align their normative beliefs closely with their empirical beliefs.

Table 2 breaks down the relative frequencies of response patterns according to the different conceptions of fairness in each social category (see columns 1 to 6). For ease of presentation, the empirical and normative beliefs of the participants in Table 2 are reported in terms of the dominant gender in a social category. In the categories CEO, firefighter, terrorist, and thief, the beliefs therefore refer to the proportion of men, and in the categories nurse and kindergartner, to the proportion of women. The breakdown of responses is based on all participants who correctly identified the dominant gender in the respective social category. In other words, all participants whose empirical belief in a category deviates in the correct direction from an equal gender distribution (i.e., “empirical belief” (E): *E > 0.5*) were included. This was the case for 94% of all participants. Roughly 4% of participants estimated the actual proportion of genders in the respective category to be equally distributed (i.e., *E = 0.5*). We excluded these participants from the present analysis because their response patterns cannot be clearly assigned to one or the other conception of fairness. For the sake of simplicity, we also excluded participants who did not correctly identify the dominant gender in a social category (i.e., *E < 0.5*). For example, participants who stated that there were more women than men in the category of CEO were excluded. However, this was the case for only 2% of participants overall. Including these participants does not change the results of the following analysis.

In columns 2 to 4 of Table 2, we break down all cases that match one of the three conceptions of fairness precisely. As described above, we base our analysis on all participants whose empirical belief matched the dominant gender in a social category (i.e., E > 0.5). If the participants’ normative belief then agreed with their empirical belief (i.e., N = E), we assigned this response pattern to proportional equality. If, instead, the participants stated that AI-generated images should depict equal numbers of men and women (i.e., *N* = 0.5), we assigned this response pattern to uniform equality. Finally, if participants stated that the dominant gender in a social category should be depicted less often than the dominated gender (i.e., *N* < 0.5), we assigned the response pattern to substantive equality. Overall, about 45% of the data matches one of the three conceptions of fairness precisely. However, uniform equality plays virtually no role in any of the six social contexts (see column 3). Only very few participants stated that AI-generated images should show gender parity between men and women if this does not correspond to reality. Slightly more participants seemed to be in favor of substantive equality (see column 4). However, by far the largest number of response patterns in all social categories were in line with proportional equality (see column 2). Many participants were apparently of the opinion that the gender distribution in AI-generated images should correspond to the respective empirical distribution.

In another 50% of response patterns, the participants’ normative and empirical beliefs were each greater than 0.5, but they did not match each other exactly. In some of these cases, the participants’ normative beliefs were smaller than their empirical beliefs (see column 5: “Underproportional”), while in others, their normative beliefs were greater than their empirical beliefs (see column 6: “Overproportional”). In a total of two-thirds of all these cases, the empirical and normative beliefs differed by exactly 10% points. Recall that participants had to specify what they considered to be the actual and appropriate proportions of men and women in a social category in increments of 10% points between 0% and 100%. Because we asked each participant to provide 15 empirical beliefs in various categories to mask the intention of our study, it was much more difficult in our experiment to respond in line with proportional equality than, for example, in line with uniform equality when specifying a normative belief in one randomly assigned social category. It is therefore reasonable to assume that some participants whose normative and empirical beliefs differed by 10% points only were actually trying to match the appropriate proportion of men and women in AI-generated images to the actual proportion in the respective social category, but forgot what they had answered at the respective question. The proportion of response patterns that corresponded precisely to proportional equality (see column 2) should therefore be regarded as the lower bound of the prevalence of proportional equality as a conception of fairness in AI-generated images among the participants.

Last but not least, we want to mention another frequent response pattern that we observed in the data. In more than 50% of the cases of “overproportional equality” (see column 6 in Table 2), the participants’ normative belief equaled 1.0. Apparently, the participants here were of the opinion that AI-generated images should mainly depict “typical” persons of a social category. These participants were presumably more concerned with the generation of paradigmatic examples in image generation than with the reproduction of the diversity within a social category. Among cases of overproportional equality, this response pattern was roughly equally prevalent in male- and female-dominated social categories (56% vs. 47%, respectively) and occurred almost equally among male and female participants (56% vs. 48%, respectively). Overall, 14% of all responses were in line with this pattern (i.e., (0.5 < E < 1.0) ∩ (*N* = 1.0)). In contrast, only 5% of all responses matched the pattern of uniform equality (i.e., (E > 0.5) ∩ (*N* = 0.5)), 12% that of substantive equality (i.e., (E > 0.5) ∩ (*N* < 0.5)) and 26% of all responses precisely matched the pattern of proportional equality (i.e., (E > 0.5) ∩ (N = E)).

**Table 2:** Individual response patterns in the data.

	E > 0.5					E ≤ 0.5
	Proport. Equal.	Uniform Equal.	Substant. Equal.	Underproport.	Overproport.	Omitted cases
	N = E	*N* = 0.5	*N* < 0.5	*N* > 0.5 N < E	*N* > 0.5 N > E	
CEO [m]	0.18	0.05	0.16	0.43	0.12	0.06
Firefighter [m]	0.32	0.04	0.10	0.14	0.37	0.03
Nurse [f]	0.26	0.09	0.12	0.29	0.18	0.06
Kindergartner [f]	0.30	0.03	0.08	0.21	0.33	0.05
Terrorist [m]	0.24	0.03	0.13	0.23	0.32	0.04
Thief [m]	0.26	0.04	0.12	0.14	0.31	0.13

*Notes*: The table shows the relative frequencies of different response patterns in all six social categories. “N” indicates normative beliefs, while “E” indicates empirical beliefs of participants regarding the appropriate or actual proportion of women or men in a social category. The empirical and normative beliefs of the participants are reported in terms of the dominant gender in a social category. The dominant gender is listed in square brackets after each social category.

Table 3 shows the results of a regression analysis in which we examined the association between participants’ normative beliefs and several other variables. The empirical beliefs, the gender of the participants, their sexist attitudes, the dominant gender, and the connotation (i.e., positive vs. negative) of the social category seemed to be obvious factors to us that could influence normative beliefs. However, as can be seen in regressions (1) and (2), among all variables, only the empirical beliefs exhibit a statistically significant association with the normative beliefs. The greater (smaller) the actual proportion of men in a social category, the greater (smaller) the proportion of men in AI-generated images should be in the corresponding category, according to the participants. From the participants’ point of view, the appropriate proportion of men and women in AI-generated images depends largely on the empirical proportion. Neither the gender of the participants nor their sexist attitudes seem to play a significant role here. The results of regression (3) also show that the association between empirical and normative beliefs does not seem to differ systematically between social categories that are either male- or female-dominated and that are either positively or negatively connoted. The positive association between empirical and normative beliefs may be a general phenomenon, independent of the specific social category.

**Table 3:** Regression analysis.

	Dependent variable: Normative belief: appropriate share of men in GenAI images		
	(1)	(2)	(3)
Constant	16.63** (7.44)	19.74*** (6.77)	21.57*** (6.97)
Empirical belief	0.70*** (0.10)	0.45*** (0.06)	0.50*** (0.15)
Participant is male (= 1)	0.81 (1.81)	1.01 (1.68)	1.22 (1.69)
Sexist attitude	− 0.03 (0.07)	− 0.05 (0.08)	− 0.17 (0.15)
Category is male-dominated (= 1)	–	17.35 (10.44)	6.22 (9.43)
Category is morally bad (= 1)	–	2.84 (9.46)	16.45 (10.52)
[Empirical belief] × [Category is male-dominated]	–	–	0.04 (0.20)
[Empirical belief] × [Category is morally bad]	–	–	− 0.17 (0.17)
[Sexist attitude] × [Category is male-dominated]	–	–	0.19 (0.18)
No. of clusters	6	6	6
Obersvations	701	701	701

*Notes*: Table 3 shows the coefficient estimates and standard errors of three regressions. The empirical and normative beliefs indicate the actual and appropriate proportion of men in a social category. The gender of the participants, whether the social category is male-dominated, and whether it has negative connotations are dummy variables. The sexist attitudes of the participants are based on the Modern Sexism Scale and vary between 8 and 56. Regressions are based on all participants who indicated whether they are male or female. Standard errors take into account the cluster structure of the data in the six social categories based on HC3 bias adjustment (see^26^.

*p < 0.05, **p < 0.01, ***p < 0.001.

## Discussion: implications and limitations

The purpose of this study was to gain a better understanding of users’ expectations regarding image generation. Only if we understand users’ expectations can we assess the alignment between image generation outputs and users’ conception of an appropriate representation. The participants prefer a representation that closely resembles their perceived empirical reality. A large group of participants even considered it more important for AI-generated images to represent stereotypical examples rather than attempting to reflect the diversity of reality. Our findings show that applying the criteria of uniform and substantive equality does not resonate with what people consider to be an appropriate representation. This is an essential finding because, while the literature discusses various fairness metrics, users’ own conceptions of appropriate representation have rarely been investigated systematically.

Participatory research seems essential to understand potential misalignments between the expectations of the developers and experts, on the one hand, and those of the broader public, on the other. Tensions may arise when such decisions are only taken by developers, i.e., big tech companies, who shape technology in accordance with their policies, as the example of Google Gemini shows, where users wanted to generate pictures of German soldiers in World War Two uniforms, and the systemʼs output was diversified regarding ethnicity^21^. Such representations are not only historically inaccurate but also morally repelling since represented minorities were not only affected by the Nazi regime but also killed by it. Neglecting user expectations and preferences or trying to educate them (here by using affirmative action policies) might have countereffects by creating more upheaval and broadening societal trenches. This suggests that companies developing AI-based systems must take such societal implications into account. This is true beyond historical cases.

One observation from our findings is that “idealistic” representations of a given social category are preferred by neither the dominant nor the marginalized groups. No systematic differences between male-dominated and female-dominated or between positively connoted or negatively connoted social roles could be observed. This suggests that users want AI representations to reflect realities as they perceive them to be.

One limitation of this study is that only one sensitive characteristic (gender) regarding social categories was tested. Further research is needed to test other attributes (such as ethnicity, disabilities, etc.) in other contexts (such as socioeconomic status). Although the present results clearly demonstrate user preference for alignment of the representation in image generation with empirical data, the normative question of what constitutes an adequate notion of fairness remains open and requires ethical deliberation beyond empirical preferences. System designers may well prioritize normative ideals over users’ preference for truthfulness to their perceived reality. However, if system designers choose to deviate from user expectations, they should make their value judgments explicit and transparent, allowing users to understand the normative commitments embedded in the system. Empirical insights into user expectations can make normative debates more productive by clarifying the actual stakes and tensions at play. In this sense, understanding what users expect and why provides a clearer foundation for ethical deliberation about appropriate fairness representations.

Moreover, reflecting on abstract principles behind different representations might alter the preferences of the users. Confronting them with such principles and their application in various contexts is an interesting line of future research that could be considered an empirical contribution to the Rawlsian idea of a “reflective equilibrium” -- the mutual adjustment between moral principles and considered judgments^27^. In terms of future research, extending the current findings by examining other sensitive attributes in certain contexts would be useful. If, as the present study suggests, usersʼ normative beliefs align with their empirical beliefs, then there is a need for research that explores the principles and whether knowledge of them could change or update the normative beliefs in specific contexts. Additionally, future research might examine potential feedback loops between AI-generated images and the formation of user expectations, as well as the influence of different modes of user engagement (affective vs. deliberate) on fairness preferences. Much work remains to be done before a full understanding of the extent of ethical implications of image generation is established.

Despite these limitations, our results suggest several important considerations for the theoretical and practical development of AI-based systems. User expectations represent a key factor to consider when developing AI-based systems for image or text generation. This seems even more pressing since studies show that the moral advice from a large language model is viewed as more trustworthy than the advice of a human expert^9^. If AI-based systems are perceived as trustworthy in moral contexts, understanding user expectations becomes crucial for an informed debate about system design, even though user preferences alone cannot determine what is ethically appropriate. Our findings suggest that users’ preferences tend to align with statistical distributions, which could serve as a potential benchmark against which such systems could be tested. However, whether and when statistical alignment should be the normative standard requires further ethical and legal deliberation. Deviations from such a standard would need to be justified explicitly, making clear which normative commitments guide the system’s design.

## Supplementary Information

Below is the link to the electronic supplementary material.

Supplementary Material 1

## Data Availability

The data will be made available upon request by the corresponding author of this publication.
