# Supplementary material for: Users prefer generative AI to reflect real over idealized gender proportions
Source: Sci Rep. 2026 Aug 11;16:24906. doi: 10.1038/s41598-026-66305-7 (PMC13462838; doi:10.1038/s41598-026-66305-7)
Supplement: Supplementary file 1 [file 41598_2026_66305_MOESM1_ESM.docx]

**Online Appendix: On-Screen Instructions**

**Screen 1:**

We will ask you some statistical questions about the proportions of certain groups in the USA, and are interested in your estimation.

**The following screens about empirical beliefs were randomized:**

How many out of 10 soccer players are left-footed?

How many out of 10 firefighters are men?

How many out of 10 cars are white?

How many out of 10 CEOs of big companies are men?

How many out of 10 thieves are men?

How many out of 10 dogs are German Shepherds?

How many out of 10 nurses are female?

How many out of 10 terrorists are male?

How many out of 10 people suffer from depression?

How many out of 10 kindergarten teachers are female?

How many out of 10 people went to college?

How many out of 10 people are left-handed?

How many out of 10 households have cats?

How many out of 10 medical doctors are female?

How many out of 10 construction workers are male?

**Screen Attention Check:**

Please select the number 4!

**Introduction to the treatments:**

In the next screen, you will see images generated by artificial intelligence. We ask you to evaluate which representation of a certain profession you find appropriate.

**We had 6 treatments, and each was also reversed, i.e., one time the number of women decreased, and in the reversed one the number of women increased. For the appendix, we selected the treatment CEO as an example.**

**Screen CEO:**

**Imagine your prompt to the AI is, "Please generate 10 pictures of CEOs." Which of the following 11 sets of pictures do you think is the appropriate representation?
------------------------------------------------------------------------------
1:**

**------------------------------------------------------------------------------
2:**

**------------------------------------------------------------------------------
3:**

**------------------------------------------------------------------------------
4:**

**------------------------------------------------------------------------------
5:**

**------------------------------------------------------------------------------
6:**
 

**------------------------------------------------------------------------------
7:**

**------------------------------------------------------------------------------
8:**

**------------------------------------------------------------------------------
9:**

**------------------------------------------------------------------------------
11:**

**------------------------------------------------------------------------------
11:**

Imagine your prompt to the AI is, "Please generate 10 pictures of CEOs." Which of the following 11 sets of pictures do you think is the appropriate representation?

Participants could select the number of the set in a dropdown menu.

**Screen for the moral evaluation of the social category on a Likert Scale from 0 to 6 (values 1 to 7):**

How do you consider the profession of "CEO" morally?

Ultimately, we asked participants some demographic questions and had them answer statements from a sexism scale.
